# Molecular Detection of Lymph Node Metastases with One-Step Nucleic Acid Amplification (OSNA) Pooling in Prostate Cancer: The POPCORN Study †

**DOI:** 10.3390/ijms252413489

**Published:** 2024-12-17

**Authors:** Mercè Cuadras, Maria E. Semidey, Jacques Planas, Inés M. de Torres, Lucas Regis, Ana Celma, Enrique Trilla, Santiago Ramón y Cajal, Rafael A. Medina, Belén Congregado, David Marcilla, Miguel A. Japón, Miguel Ramirez, Ana Calatrava-Fons, Asier Leivar, María B. Alonso, Eugenia García, Pilar González-Peramato, Dario Vazquez-Martul, Ángel Concha-López, Venancio Chantada, Francisco J. Queipo, José L. Gago, Cristina Carrato, Rafael J. Luque, Juan Moreno-Jimenez, Inmaculada Catalina-Fernández, Cristina León, Juan Morote

**Affiliations:** 1 Urology Department, Vall d’Hebron University Hospital, 08035 Barcelona, Spain; jacques.planas@vallhebron.cat (J.P.); lucas.regis@vallhebron.cat (L.R.); ana.celma@vallhebron.cat (A.C.); enrique.trilla@vallhebron.cat (E.T.); juan.morote@uab.cat (J.M.); 2 Department of Surgery, Universitat Autònoma de Barcelona, 08193 Bellaterra, Spain; 3 Pathology Department, Vall d’Hebron University Hospital, 08035 Barcelona, Spain; maru.semidey@vallhebron.cat (M.E.S.); ines.detorres@vallhebron.cat (I.M.d.T.); santiago.ramonycajal@vallhebron.cat (S.R.y.C.); 4 Department of Morphological Sciences, Universitat Autónoma de Barcelona, 08193 Bellaterra, Spain; 5 Urology Department, Hospital Universitario Virgen del Rocío, 41013 Sevilla, Spain; rantonio.medina.sspa@juntadeandalucia.es (R.A.M.); belencongregadoruiz@gmail.com (B.C.); 6 Pathology Department, Hospital Universitario Virgen del Rocío, 41013 Sevilla, Spain; david.marcilla.sspa@juntadeandalucia.es (D.M.); mangel.japon.sspa@juntadeandalucia.es (M.A.J.); 7 Clínica Urosalud, 46021 Valencia, Spain; mramirezb@clinica-urosalud.es; 8 Pathology Department, Institut Valencià d’Oncologia, 46009 Valencia, Spain; acalatrava@fivo.org; 9 Urology Department, Hospital Universitario La Paz, 28046 Madrid, Spain; asier.leivar@salud.madrid.org (A.L.); mb.alonsobartolome@gmail.com (M.B.A.); 10 Pathology Department, Hospital Universitario La Paz, 28046 Madrid, Spain; egfernadez@salud.madrid.org (E.G.); mpilar.gonzalezperamato@salud.madrid.org (P.G.-P.); 11 Urology Department, Hospital Universitario A Coruña, 15006 A Coruña, Spain; dario.martul@gmail.com (D.V.-M.); venancio.chantada.abal@sergas.es (V.C.); 12 Pathology Department, Hospital Universitario A Coruña, 15006 A Coruña, Spain; angel.concha.lopez@sergas.es (Á.C.-L.); patxiqueipo@gmail.com (F.J.Q.); 13 Urology Department, Hospital Universitari Germans Trias i Pujol, 08916 Badalona, Spain; jlgago.germanstrias@gencat.cat; 14 Pathology Department, Hospital Universitari Germans Trias i Pujol, 08916 Badalona, Spain; ccarrato.germanstrias@gencat.cat; 15 Pathology Department, Hospital Universitario de Jaén, 23007 Jaén, Spain; rafaelj.luque.sspa@juntadeandalucia.es; 16 Urology Department, Hospital Universitario de Jaén, 23007 Jaén, Spain; juanmoreno35@hotmail.com; 17 Pathology Department, Hospital Universitario de Puerto Real, 11510 Cádiz, Spain; mycafe@telefonica.net; 18 Urology Department, Hospital Universitario Puerta del Mar, 11510 Cádiz, Spain; cristinaleondelgado@hotmail.com

**Keywords:** prostate cancer, pelvic lymph node dissection, lymph node metastases, OSNA, cytokeratin 19, pooling, hematoxylin and eosin staining

## Abstract

Pelvic lymph node dissection (PLND) is the most accurate procedure for lymph node (LN) staging in prostate cancer (PCa) patients. LN sectioning and hematoxylin and eosin (H&E) staining of at least one slice remains the gold standard for LN evaluation, potentially leading to misdetection of small metastatic focus. Entire LN analysis is possible with One-Step Nucleic Acid Amplification (OSNA) by detecting cytokeratin 19 (CK19) mRNA as a surrogate for LN invasion. This study aimed to compare postoperative performance of OSNA pooling with conventional H&E staining for pathological LN detection in PCa patients. POPCORN was an observational, prospective, and multicenter study of patients with PCa who underwent PLND. Dissected LNs were analyzed by both methods. This study included 2503 LNs from 131 patients, showing no statistically significant differences in pathological LN detection. Concordance between methods was high (93.9%), as were specificity (96.6%) and negative predictive value (96.6%) of OSNA pooling. The measure of agreement (Cohen’s Kappa [κ]) was 0.70. Only eight (6.1%) discordances were observed, including four misdetections from each method. Results showed a high concordance between OSNA pooling and H&E staining, suggesting that OSNA pooling may be a good alternative to H&E staining to detect LN metastases in PCa patients.

## 1. Introduction

Pelvic lymph nodes (LNs) are the most frequent site of metastases in prostate cancer (PCa), with 13.8% to 18.8% of patients with PCa who underwent radical prostatectomy with pelvic LN dissection (PLND) showing LN invasion [1,2]. In addition, pelvic LN metastases have been related to worse prognoses [3,4,5]. Therefore, identifying pelvic LN metastases is crucial to guide therapeutic decisions in these patients.

To date, no presurgical test has shown enough sensitivity to detect LN metastases [6]. In recent years, new imaging techniques have been developed for diagnosing LN invasion in patients with PCa, such as Ga-68 prostate-specific membrane antigen (PSMA) positron emission tomography combined with computed tomography (PET/CT) scan, which has shown improved sensitivity and accuracy compared with conventional imaging techniques [7]. However, Ga-68 PSMA-PET/CT scan does not detect most of the smallest metastatic LNs (i.e., <5 mm) compared with histopathological examination [8]. Additionally, according to recent findings, PSMA-PET/CT scan does not preclude the need for an extended PLND in PCa patients at high risk or in those with ductal adenocarcinoma [9]. Thus, extended PLND remains the most accurate method for LN staging of patients with localized and locally advanced PCa. European Association of Urology (EAU) risk groups for biochemical recurrence [10] and nomograms are used to assess the risk of having LN metastases and consequently identify candidates for extended PLND [11].

After performing a PLND, LNs are sectioned into 3–4 mm slices, and at least one of them is prepared for microscopic examination after hematoxylin and eosin (H&E) staining [12]. This technique is currently the gold-standard method for LN assessment, but also presents several limitations, including low sensitivity in detecting micrometastases—generally due to tissue allocation bias (TAB), which hinders the detection of metastases located outside the analyzed slice—interobserver bias, and its time-consuming nature. To overcome these limitations, new molecular methods have been developed, such as One-Step Nucleic Acid Amplification (OSNA).

OSNA is a molecular technique that allows the detection of mRNA of cytokeratin 19 (CK19), an epithelial membrane protein found in some tumors and used as a biomarker of LN metastases in different types of cancer [13,14]. It allows a quick analysis of the entire LN, either intraoperatively or in a postoperative scenario, by using the reverse-transcription loop-mediated isothermal amplification (RT-LAMP) method to amplify CK19 mRNA. This technique was initially applied for the intraoperative evaluation of sentinel LNs in breast [15], but it also allows a pooled analysis of a high number of LNs with few molecular tests, as demonstrated for patients with colorectal carcinoma or non-small cell lung cancer [16,17].

Regarding PCa, Winter et al. detected the overexpression of CK19 mRNA with OSNA in primary PCa tumors of all patients included in the study [18]. Later, Engels et al. used OSNA to stage 64 patients with PCa, finding a good sensitivity, specificity, and concordance versus H&E staining [19]. However, more studies are needed to confirm the usefulness of OSNA in staging PCa patients. Given the lack of consensus on defining and identifying sentinel LNs in PCa, the current guidelines’ recommendations on conducting an extended PLND when LN staging is required, and the high yield of LNs retrieved following an extended PLND, we considered it essential to evaluate the performance of the OSNA technique for identifying LN metastases by using a LN pooling approach. Additionally, we hypothesized that LN staging via OSNA pooling may be at least comparable to LN staging performed with H&E staining. Therefore, this study aimed to assess the performance of OSNA (specifically, OSNA pooling) in detecting LN metastases in patients with PCa who had undergone PLND using conventional H&E staining as the reference method.

## 2. Results

### 2.1. Characteristics of Study Patients

This study included 131 patients, of whom only 1 (0.8%) had undergone a salvage PLND. As shown in Table 1, at the time of diagnosis, most patients were classified as having PCa at clinical stages T1c/2a and N0, and the most common International Society of Urological Pathology (ISUP) grade groups of biopsies were 2 and 3. Similarly, after surgery, most patients were diagnosed with pathological stages T2/3a and N0, and the most common ISUP grade groups of the surgically removed prostates were 2 and 3.

A total of 2503 LNs were removed, corresponding to a median (IQR) of 18.0 (14.0–23.0) LNs per patient. According to H&E staining, 116 (88.6%) patients did not have any pathological LN, 11 (8.5%) showed 1 or 2 metastatic LNs, and 4 (3.0%) had >2 LNs affected.

### 2.2. Detection of Lymph Node Metastases per Patient with OSNA Pooling

**Figure 1:**
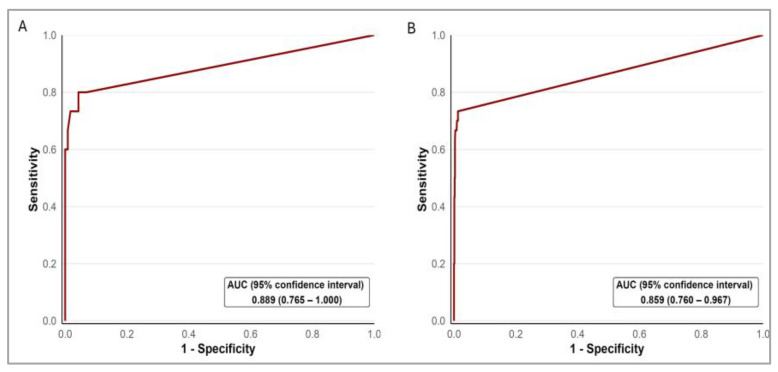
Receiver operating characteristic curves showing the discrimination ability of OSNA to identify lymph node metastases already diagnosed with H&E staining. Results are presented by patient (**A**) and sample tube (**B**). A threshold of 250 copies/μL of CK19 mRNA is used as a surrogate marker for LN metastasis in PCa, as outlined in the manufacturer’s manual.

After LN sample pooling, 972 sample tubes were obtained to be analyzed by OSNA pooling, containing a median (IQR) of 2.0 (1.0–3.0) LNs each. The discrimination capacity of this method to identify LN metastases already detected with H&E staining is shown in Figure 1.

**Figure 2:**
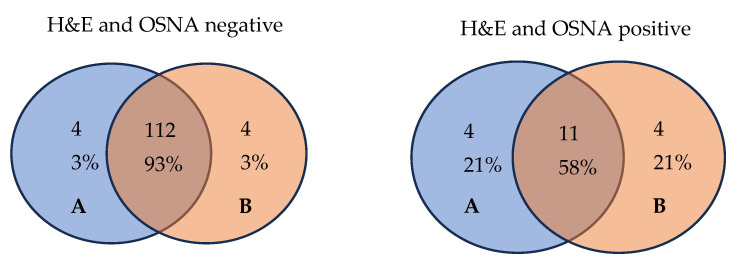
Comparison of examination results for the presence of tumor cells using the H&E staining and OSNA pooling methods. A: H&E, hematoxylin and eosin; B: OSNA, One-Step Nucleic Acid Amplification.

As seen in Table 2, no statistically significant differences were observed between OSNA pooling and conventional H&E staining for LN metastasis detection, and only eight (6.1%) patients showed discordant results with H&E staining and OSNA pooling: four (3.1%) patients tested positive according to OSNA pooling but negative as per H&E staining, and four (3.1%) patients tested negative according to OSNA pooling but positive as per H&E staining. The Venn diagram represented in Figure 2 visually delineates the overlap and differences in results between H&E staining and OSNA pooling.

Additionally, Table 3 shows a strong concordance between OSNA pooling and conventional H&E staining, a high level of agreement, and robust specificity and NPV for OSNA.

### 2.3. Discordances Between H&E Staining and OSNA Pooling

**Figure 3:**
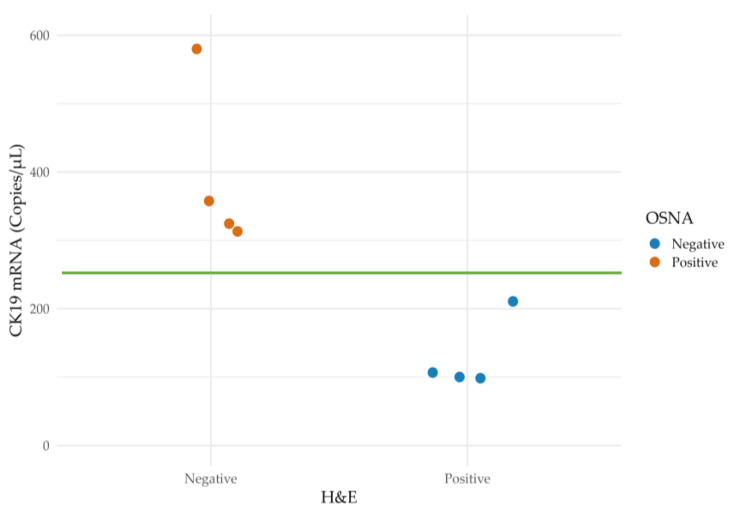
Scatter plot representing CK19 mRNA levels in discordant cases. The green line marks the cut off of 250 copies/μL of CK19 mRNA, above which the case is considered positive or pathological. Note that the three discordant cases located below 160 copies/μL are represented in the minimum established detection value of OSNA. CK19, cytokeratin 19; H&E, hematoxylin and eosin; OSNA, One-Step Nucleic Acid Amplification.

Figure 3 shows an overview of the concordance between OSNA and histopathology considering the copies of CK19 mRNA detected by OSNA.

Table 4 provides details of the eight discordant cases. In all of these, the same results were obtained after applying the respective confirmation techniques. In the four cases where OSNA pooling tested positive (but H&E staining tested negative), micrometastases were identified. Conversely, among the four patients where H&E staining was positive (but negative according to OSNA pooling), two were micrometastases and the other two. macrometastases. Notably, one of the micrometastases identified exclusively by H&E staining showed a CK19 mRNA total tumor load (TTL) of 210 copies/μL in the OSNA pooling analysis. Table 5 shows additional information regarding the discordant cases.

## 3. Discussion

In this study of patients with PCa and indication of PLND, LN invasion was found to be present in 15 (11.5%) patients according to H&E staining, and in 15 (11.5%) patients according to OSNA pooling, although eight (6.1%) discordant cases were observed. No statistically significant differences were observed in the results between OSNA pooling and conventional H&E staining for LN metastasis detection. In addition, the concordance between both methods was very high (93.9%), as well as the specificity (96.6%) and NPV (96.6%) of OSNA pooling.

To our knowledge, only two studies have compared H&E staining and OSNA for the detection of pelvic LN metastases in PCa patients [19,21]. Among them, the study by Engels et al. also evaluated the performance of OSNA on a per-LN basis, reporting concordance, sensitivity, and specificity values of 94.4%, 84.2%, and 96.6%, respectively [19]. These results are consistent with our patient-based findings, which showed concordance, sensitivity, and specificity of 93.9%, 73.3%, and 96.6%, respectively.

However, the study of Engels et al. [19] and ours show some differences. Engels et al. included 64 PCa patients who underwent extended and magnetometer-guided sentinel PLND, examining only sentinel LNs (*n* = 534), whereas, in our study, all dissected LNs (*n* = 2503) obtained from PLND of 131 PCa patients were analyzed. While an OSNA pooling approach was conducted in our study, Engels et al. performed an OSNA analysis for each individual sentinel LN. CK19 expression in primary tumors was stated as an inclusion criterion in our study. In contrast, Engels et al. assessed CK19 expression in primary tumors in the discordant cases, identifying a low or no expression of CK19 in five of six OSNA-negative macrometastases cases, and suggesting the importance of CK19 determination at diagnosis. These differences may explain the variation in sensitivity among the two studies. Moreover, based on recent literature, a lower rate of positive LNs after PLND than expected was found, which may result from a higher proportion of intermediate versus high-risk population in our study. A higher number of patients with LN metastases may have improved our OSNA pooling sensitivity value.

Regarding sentinel LNs in PCa, the recent prospective SENTINELLE study showed good performance of intraoperative gamma probe-guided sentinel LN biopsy compared to extended PLND as reference standard for LN staging in patients with intermediate or high risk localized PCa patients [22]. However, there is still a lack of consensus on the definition and identification of sentinel LN in PCa, and current guidelines strongly recommend an extended PLND when LN staging is mandatory [10]. Thus, as conducted in other types of tumors when lacking a clear sentinel LN definition [16,17], LNs were analyzed by OSNA pooling, which, to our knowledge, has not been reported yet in PCa patients. Nevertheless, if sentinel LNs become well defined for PCa in the future, we believe that OSNA may be used intraoperatively to identify sentinel LN invasion, as in patients with breast cancer [23]. This approach may help avoid extended PLND and its associated morbidity, and the lower number of LNs removed would decrease surgical time and pathologists’ burden.

As opposed to the OSNA procedure conducted by Engels et al. [19] and H&E staining, the OSNA pooling method applied in this study prevented us from determining the number of affected LNs per patient, which should be considered to decide subsequent treatments, such as adjuvant therapy. Further, it allowed us to assess the TTL of patients while entailing lower costs than performing OSNA for each individual LN. Moreover, OSNA represents a standardized, fast, reproducible and objective method that can analyze the whole LNs. In addition, using molecular techniques instead of histopathological assessments seems to enhance the detection of LN micrometastases in PCa patients [24].

In our study, eight discordant cases were identified, six of which were micrometastases. These discrepancies may be attributed to TAB, where metastases were present in LN samples assessed by H&E staining but absent in those analyzed using OSNA assay, or vice versa. Furthermore, the use of different LN sections for different procedures has been described as the main reason for discrepancies between molecular and histological analyses [19,25]. The remaining two cases, which involved macrometastasis detected solely by H&E staining, may also be explained by TAB. However, an alternative explanation for these two discordances could be the heterogeneous expression of cytokeratin 19 (CK19), as previously reported, which varies according to the degree of cancer dedifferentiation [26]. In this regard, we tried to avoid cases with a weak CK19 mRNA expression by using positive CK19 immunohistochemistry in the biopsy of the primary tumor as one of the inclusion criteria of our study. Nevertheless, when revising the results of the pathology department, we found that almost all metastases only detected by H&E staining corresponded to cases with prognostically poor criteria according to Engels et al. (i.e., pathological stage T3, high tumor volume, ISUP grade ≥ 3, poor Gleason grading) [19]. Therefore, it is possible that cases with advanced and dedifferentiated tumors are not detectable by OSNA due to a lack of CK19 mRNA expression [26], although Winter et al. demonstrated CK19 mRNA expression in 100% of the PCa specimens analyzed by OSNA, regardless of the degree of tissue dedifferentiation. More studies are needed to address the variability in CK19 mRNA expression in PCa, as well as to consider the possibility of analyzing other PCa-specific biomarkers, such as PSMA [27], either alone or in combination with CK19. Additionally, one micrometastasis detected exclusively by H&E staining produced a CK19 mRNA TTL of 210 copies/μL by OSNA pooling, which is close to the positivity threshold of ≥250 copies/μL for OSNA. This finding raises the question of whether this borderline value holds clinical significance in PCa, although further studies are required to set a specific cut-off point for PCa.

This study presents some limitations. As mentioned, the OSNA pooling approach prevented us from assessing LN morphological aspects and the number of affected LNs per patient, which may be considered for assessing the need for adjuvant therapy according to current PCa guidelines [10]. Nonetheless, it did allow us to determine the TTL per patient, which may have prognostic and therapeutic significance in the future, as described in studies with breast cancer patients [28,29,30]. Methodologically, the need to split the LN entailed risk of TAB, which might have affected the comparison between both techniques. However, the large number of LNs in the study may have helped to overcome this drawback. Despite these limitations, to our knowledge, this is the first study comparing H&E staining and OSNA pooling method for identifying LN metastases in PCa patients.

Next steps to consolidate the clinical application of OSNA in PCa might come from further well-designed prospective and randomized studies, so that LN is not divided, and TAB can be avoided. Specific cut-off points or even the prognostic impact of TTL value for the management of these patients might be explored. Additionally, the cost-effectiveness of the OSNA pooling method should be addressed in future studies to confirm if introducing this approach can both economically and clinically benefit healthcare providers and patients, respectively, as previously reported for patients with colorectal carcinoma [31].

## 4. Materials and Methods

### 4.1. Study Design and Patients

POPCORN was an observational, prospective, and multicenter study of patients with PCa and indication of PLND. This study was performed in eight tertiary centers from Spain between September 2019 and June 2023.

Patients were included in this study if they had been diagnosed with primary, histologically confirmed PCa, showed positive CK19 immunohistochemistry (i.e., ≥50% of membrane or cytoplasmic staining) in a biopsy specimen core, and had an indication of radical prostatectomy with extended PLND. Only patients with a minimum of 12 LNs removed were included, as previously established for standard (10–12 LNs) and extended (>12 LNs) PLND [32,33].

Alternatively, they could have undergone salvage PLND due to biochemical recurrence and present LN uptake by choline PET scan. Patients with metastatic PCa or other CK19-positive tumors with the same lymphatic drainage and patients receiving or having received neoadjuvant therapies were excluded from this study.

All patients had their LNs analyzed by OSNA pooling and H&E staining. However, all therapeutic decisions were based only on the results obtained by the conventional method (i.e., H&E staining), as normally performed in all the participant centers.

All data were processed according to General Data Protection Regulation 2016/679 on data protection and privacy for all individuals within the European Union and the local data protection regulatory framework [34]. This study was approved by ethics committee at the coordinating center, Ethics Committee of Hospital Vall d’Hebron (PR(AG)316/2019), and all the local ethics committees.

### 4.2. Study Variables

Age, serum prostate-specific antigen (PSA), clinical T stage by rectal digital examination and clinical N stage by computed tomography scan [20], prostate biopsy grade group according to the ISUP grading system [35], and the criteria used to determine the indication for lymphadenectomy were collected at the time of diagnosis. In addition, the number of LNs surgically removed, the pathological T and N stages [20], and the surgical specimen grade group [35] were recorded postoperatively. Other variables registered included the tumor volume and Gleason patterns.

### 4.3. Collection and Processing of Lymph Nodes

Samples were collected from 130 patients who underwent radical prostatectomy with an extended PLND, which included all LNs overlying the external iliac artery and vein, those within the obturator fossa located cranially and caudally to the obturator nerve, and those medial and lateral to the internal iliac artery, as described in the PCa European Guidelines [10]. One patient with indication of salvage PLND was submitted to a more extended surgery, including common iliac artery and presacral LNs.

**Figure 4:**
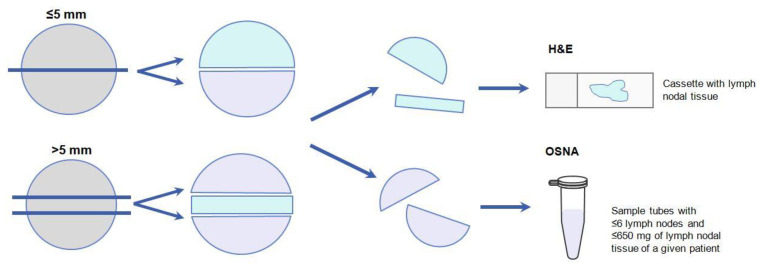
Processing of lymph nodes for hematoxylin and eosin (H&E) staining and One-Step Nucleic Acid Amplification (OSNA) pooling analyses.

Once removed, LNs were immediately sent to the pathology department, where they were dissected from the adipose tissue on an ice-cooled surface. Then, to reproduce the conventional diagnosis by H&E staining, in which a central slice of 3–4 mm of the LN is usually analyzed, each LN was cut along its long axis. All LNs were analyzed by H&E staining and OSNA pooling. For this purpose, LNs ≤ 5 mm were cut into two halves, and each half was analyzed using the above methods. One half was put into a cassette for standard H&E, and the other half was introduced into a microcentrifuge tube for delayed OSNA analysis. For LNs > 5 mm, a central slice of approximately 2 mm was cut. The central slice was examined using standard H&E staining, and both lateral slices were submitted for OSNA analysis (Figure 4).

When discordant results were obtained, additional assessments were conducted. In case an LN did not show metastasis as per H&E staining, but it did as per OSNA, the remaining LN tissue embedded in paraffin was divided into three levels, and two samples were collected in each level; one sample was used for another assessment with H&E staining, whereas the other one was used for an evaluation by CK19 immunohistochemistry. In case a LN showed metastasis as per H&E staining but not as per OSNA, the OSNA procedure was repeated for the sample tube where discordant results were found.

### 4.4. H&E Staining

The formalin-fixed LN tissue was embedded in paraffin and cut into 5 μm-thick sections. After deparaffinization and rehydration, these sections were automatically stained with H&E. According to UICC TNM 8th edition [20], micrometastasis was defined as LN metastatic focus between 0.2 and 2 mm, whereas a metastatic focus of more than 2 mm was considered a macrometastasis.

### 4.5. OSNA Pooling Analysis

The analysis of LNs by OSNA was performed with pooled samples of a given patient. For this purpose, LNs were put together into microcentrifuge tubes, each containing a maximum of six LNs, which added up to 650 mg. Each tube had a paraffin block embedding multiple LNs. LNs weighing >650 mg were cut into fragments ≤650 mg and placed in different microcentrifuge tubes. The tubes were then stored at −80 °C for ulterior OSNA analysis, which was conducted using the RD-210 platform (Sysmex Corporation, Kobe, Japan) following the manufacturer’s instructions.

After OSNA analysis and according to the threshold reported by Tsujimoto et al. [15] for detecting LN metastases in breast cancer, sample tubes and patients were defined as negative or positive when CK19 mRNA levels were <250 copies/μL or ≥250 copies/μL, respectively. Additionally, the TTL for a given patient was defined as the sum of all CK19 mRNA copies corresponding to all sample tubes with CK19 mRNA overexpression (i.e., ≥250 copies/μL). Micro- and macrometastases were defined as proposed by Tsujimoto et al. for breast cancer (i.e., micrometastases when CK19 mRNA levels ranged between 250 and 4999 copies/μL; and macrometastases when CK19 mRNA levels were ≥5000 copies/μL) [15].

### 4.6. Statistical Analysis

The statistical analysis was performed using R Studio version 4.3.2. Main clinical and pathological data of interest were reported. Quantitative variables were described as the median and interquartile range (IQR, 25th and 75th percentiles), whereas qualitative variables were presented as frequencies and percentages. To assess the discrimination ability of OSNA to identify LN metastases already detected with H&E staining, receiver operating characteristic (ROC) curves were plotted, and the corresponding area under the curve (AUC) was determined. To compare the performance of OSNA and conventional H&E staining in detecting LN metastases, crosstabs and McNemar’s test were used, and concordance and the measure of agreement (Cohen’s Kappa coefficient [κ]) between the two methods was determined. Sensitivity, specificity, and positive and negative predictive values (PPV and NPV) of OSNA were also calculated. The threshold for statistical significance was set at a two-sided alpha value of 0.05.

## 5. Conclusions

The OSNA pooling method was comparable to H&E staining for the detection of LN invasion after PLND in PCa patients.

This article is a revised and expanded version of a paper entitled “POPCORN Study: Application of the “One-Step Nucleic Acid Amplification” (OSNA) Method for the Detection of Nodal Metastasis in Patients with Prostate Cancer”, which was presented at the Congress of the European Association of Urology (EAU24), Paris (France), 7 April 2024 [36].

## Figures and Tables

**Table 1:** Characteristics of study patients at diagnosis. *n* = 131, unless otherwise specified.

Age (years), median (IQR)	65.0 (59.5–69.0)
Serum PSA (ng/mL), median (IQR)	8.5 (5.7–12.7)
Clinical T stage, *n* (%), *n* = 130	
cT1c	76 (58.5)
cT2a	28 (21.5)
cT2b	12 (9.2)
cT2c	5 (3.8)
cT3	9 (6.9)
Clinical N stage, *n* (%)	
0	126 (96.2)
1	5 (3.8)
Prostate biopsy grade group, *n* (%), *n* = 130	
1	10 (7.7)
2	37 (28.5)
3	61 (46.9)
4	22 (16.9)
5	0 (0.0)
Criteria for lymphadenectomy, *n* (%), *n* = 130	
Intermediate risk (nomogram)	92 (70.8)
High risk	38 (29.2)
Pathological T stage, *n* (%), *n* = 129	
pT2	48 (37.2)
pT3a	54 (41.9)
pT3b	27 (20.9)
Pathological N stage, *n* (%)	
0	116 (88.6)
1	15 (11.5)
Surgical specimen grade group, *n* (%), *n* = 129	
1	1 (0.8)
2	42 (32.6)
3	67 (51.9)
4	12 (9.3)
5	7 (5.4)

PSA, prostate-specific antigen.

**Table 2:** Crosstabs of OSNA pooling results per patient, using H&E staining as reference method, *n* (%).

		H&E Results			*p*
		Negative	Positive	All	
OSNA results	<250 copies	112 (85.5)	4 (3.1)	116 (88.5)	1.00
	≥250 copies	4 (3.1)	11 (8.4)	15 (11.5)	
	All	116 (88.5)	15 (11.5)	131 (100.0)	

H&E, hematoxylin and eosin; OSNA, One-Step Nucleic Acid Amplification.

**Table 3:** Concordance and performance parameters of OSNA pooling results to identify lymph node metastases per patient using H&E staining as reference method, % (95% confidence intervals).

Concordance	93.9 (88.4–96.9)
Sensitivity	73.3 (48.1–89.1)
Specificity	96.6 (91.5–98.7)
PPV	73.3 (48.1–89.1)
NPV	96.6 (91.5–98.7)
Kappa coefficient	0.70 (0.62–0.78)

H&E, hematoxylin and eosin; OSNA, One-Step Nucleic Acid Amplification; NPV, negative predictive value; PPV, positive predictive value.

**Table 4:** Detailed analysis of cases with discordant results with H&E staining and OSNA pooling techniques.

Patient ID	H&E Results (Number of Positive LNs)	OSNA Results (CK19 mRNA Copies/μL)	Type of Metastasis * from Positive Results	Supplementary Tests and Results
2	Negative (NA)	Positive (360)	Micrometastasis	CK19 IHC: negative
13	Negative (NA)	Positive (580)	Micrometastasis	CK19 IHC: negative
99	Negative (NA)	Positive (330)	Micrometastasis	CK19 IHC: negative
100	Negative (NA)	Positive (310)	Micrometastasis	CK19 IHC: negative
7	Positive (2)	Negative (210)	Micrometastasis	OSNA repetition: negative (210 copies/μL)
74	Positive (2)	Negative (<160)	Macrometastasis	OSNA repetition: negative (<160 copies/μL)
101	Positive (2)	Negative (<160)	Macrometastasis	OSNA repetition: negative (<160 copies/μL)
121	Positive (1)	Negative (<160)	Micrometastasis	OSNA repetition: negative (<160 copies/μL)

* Micro- and macrometastases were defined according to LN metastatic focus size (0.2–2 mm, micrometastasis; >2 mm, macrometastasis [20]) (H&E staining) or CK19 mRNA levels (250–4999 copies/μL, micrometastasis; ≥5000 copies/μL, macrometastasis as proposed by Tsujimoto et al. for breast cancer [15]) (OSNA pooling). CK19, cytokeratin 19; H&E, hematoxylin and eosin; IHC, immunohistochemistry; NA, not applicable; OSNA, One-Step Nucleic Acid Amplification.

**Table 5:** Characteristics of the primary tumors of discordant cases.

Patient ID	Pathological T Stage	Tumor Volume	ISUP Grade	% of Gleason Patterns ≥ 3
2	2	Low	3	60% (pattern 4)
13	3a	Low	2	40% (pattern 4)
99	3a	Low	3	60% (pattern 4)
100	3a	High	3	70% (pattern 4) + 3% (pattern 5)
7	3b	High	3	90% (pattern 4)
74	3a	High	5	50% (pattern 4) + 40% (pattern 5)
101	3a	Low	2	10% (pattern 4)
121	3a	High	3	70% (pattern 4)

H&E, hematoxylin and eosin; ISUP, International Society of Urological Pathology; OSNA, One-Step Nucleic Acid Amplification.

## Data Availability

The data that support the findings of this study are available from the corresponding author upon reasonable request.
